# Mini-review: microbiota have potential to prevent PEDV infection by improved intestinal barrier

**DOI:** 10.3389/fimmu.2023.1230937

**Published:** 2023-07-12

**Authors:** Shanshan Yang, Guangliang Liu, Huub F. J. Savelkoul, Christine A. Jansen, Bin Li

**Affiliations:** ^1^ Key Laboratory of Veterinary Biological Engineering and Technology, Institute of Veterinary Medicine, Jiangsu Academy of Agricultural Sciences, Ministry of Agriculture, Nanjing, China; ^2^ Jiangsu Key Laboratory for Food Quality and Safety-State, Institute of Veterinary Medicine, Jiangsu Academy of Agricultural Sciences, Ministry of Agriculture, Nanjing, China; ^3^ Key Laboratory Cultivation Base of Ministry of Science and Technology, Institute of Veterinary Medicine, Jiangsu Academy of Agricultural Sciences, Ministry of Agriculture, Nanjing, China; ^4^ Jiangsu Co-innovation Center for Prevention and Control of Important Animal Infectious Diseases and Zoonoses, Yangzhou University, Yangzhou, China; ^5^ State Key Laboratory of Animal Disease Control and Prevention, College of Veterinary Medicine, Lanzhou University, Lanzhou Veterinary Research Institute, Chinese Academy of Agricultural Sciences, Lanzhou, Gansu, China; ^6^ Cell Biology and Immunology Group, Wageningen University and Research, Wageningen, Netherlands

**Keywords:** intestinal microbes, PEDV infection, mucosal barrier, interaction, antivirus, piglets

## Abstract

Porcine epidemic diarrhea virus (PEDV) infection poses a significant threat to the global pig industry. Current prevention and control strategies are inadequate in protecting pigs from new PEDV variants. This review aims to examine the relationship between PEDV and intestinal microbes, and investigate whether modulating intestinal microbes could affect PEDV infection. The mechanisms by which various intestinal microbes affect viral infection were initially introduced. Intestinal microbes can influence enteric viral infection through direct contact, such as binding, or by affecting interferons (IFNs) production and the intestinal barrier. Influencing the intestinal barrier by microbes can impact PEDV infection in young piglets. To narrow down the range of microbes that may influence PEDV infection, this review summarized microbes that change after infection. Short chain fatty acids (SCFAs), bacterial cell components, and toxins from microbes were identified as important mediators affecting PEDV infection. SCFAs primarily strengthen the intestinal barrier and inhibit intestinal inflammation, while bacterial cell components and toxins are more likely to damage the intestinal barrier. Therefore, this review hypothesizes that fecal transplantation, which allows the host to colonize more SCFAs-producing microbes, may prevent PEDV infection. However, these hypotheses require further proof, and the transplantation of intestinal microbes in pigs requires more exploration.

## 1 Introduction

Porcine epidemic diarrhea (PED) is a highly infectious disease that affects pigs, and is caused by the porcine epidemic diarrhea virus (PEDV) (1). The recurring outbreaks of PED in both China and the US since 2010 indicate that the current vaccines and antiviral drugs are ineffective in preventing infections caused by newly evolved and highly pathogenic PEDV variants (2, 3). Currently, the global pig industry remains threatened by PED, which continues to be one of the most significant infectious diseases. PEDV infection causes severe damage to the intestinal barrier in the small intestine, leading to watery diarrhea, vomiting, dehydration, and 100% mortality in piglets younger than one week of age (4).

**Figure 1:**
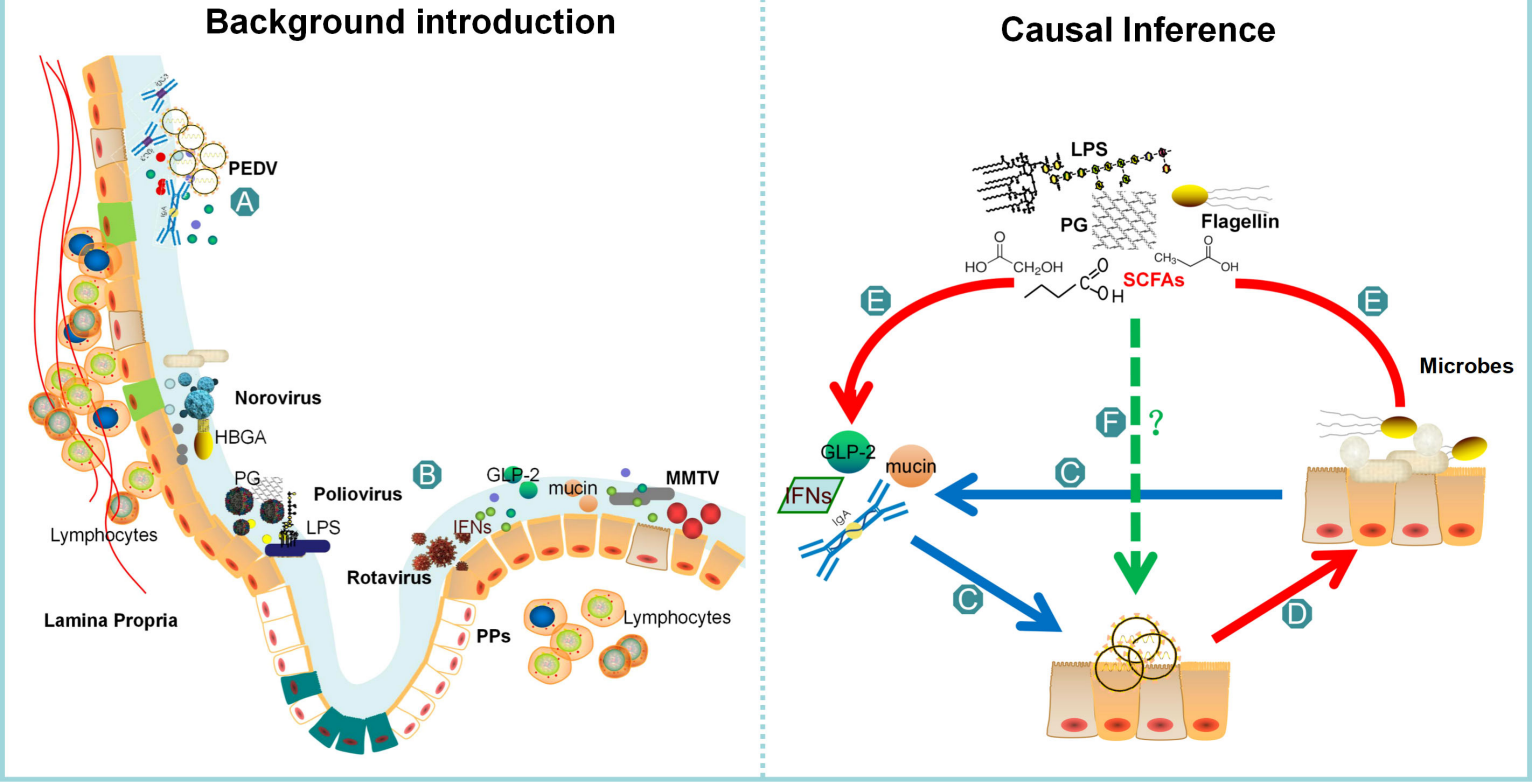
The comprehensive structure of this review. The background of PEDV is presented as **(A)**, while **(B)** highlights the current report on all microbial groups that affect virus invasion. Bacteria can influence viral infection through direct actions such as binding and adhesion, or indirectly by affecting interferons or the intestinal barrier. The intestinal barrier, represented as **(C)**, plays a crucial role in regulating PEDV infection, and some bacteria or their metabolites can affect its function. The review summarizes the intestinal microbes remodeled by PEDV infection as **(D)**, which explains that the number of probiotics decreases while the number of pathogenic bacteria increases. **(E)** highlights the common feature of the changing microbes, which can regulate interferons and the integrity of the intestinal barrier. Finally, in **(F)** part, the review concludes that further verification is necessary to determine whether these microbes or metabolites can affect PEDV infection.

The intestinal barrier comprises a range of epithelial cells such as stem cells, Paneth cells, goblet cells, tuft cells, enteroendocrine cells, enterocytes, and microfold cells, and serves as a crucial component of the intestinal microenvironment (5). The upper layer of the barrier is coated with a mucus layer housing different microbes. Beneath the epithelial cells lies the lamina propria, which is home to various immune cells such as macrophages, dendritic cells, T cells and B cells (**Figure 1A**) (6). Within the intestinal microenvironment, microbes hold a significant position in maintaining intestinal homeostasis and combating viral infections (7, 8). This review elucidates the capacity of intestinal microbes to regulate viral infection through various mechanisms, thereby implying the prospect of manipulating microbial composition as a means of inhibiting PEDV infection.

## 2 Intestinal microbes influence various viral infection

Intestinal microbes enhance the infection of various viruses, including norovirus, poliovirus, mouse mammary tumor virus (MMTV), and rotavirus (**Figure 1B**) (9). This facilitation of viral infection by intestinal microbes is partly due to their direct contact with the virus, which increases viral virulence. *In vitro* studies have shown that the presence of *Enterobacter. cloacae* expressing Histo-blood group antigens (HBGA) is required for the infection of B cells with human norovirus (10, 11). Furthermore, the mucin-binding ability of HBGA-expressing *E. coli* increases viral infectivity under acute heat stress. When noroviruses are mixed with HBGA-expressing *E. coli*, their antigen integrity is less likely to be destroyed even when heated at 90°C for 2 minutes *in vitro* (12). Transmission electron microscopy has demonstrated that norovirus-like particles also bind to extracellular polymeric substances of *Enterobacter* sp. *SENG-6*, where the HBGAs are localized (13). Additionally, when co-infected with norovirus, *L. johnsonii* aids in the genetic recombination of the virus, causing the removal of mutations in drug or temperature sensitive genes. This process restores viral fitness and enhances its ability to infect (9). Other components of enteric bacteria, such as the outer cell membrane and pili, are able to bind norovirus and increase the viral infection rate (14). Moreover, the presence of *Bacillus cereus* has been shown to significantly enhance poliovirus adherence and increase viral PFU by up to 500% in HeLa cells compared to a control group without bacteria. Interestingly, this heightened infection rate is not contingent on the presence of live bacteria, nor is it dependent on any cellular effects. Rather, bacterial surface polysaccharides, specifically lipopolysaccharide (LPS) and peptidoglycan (PG), are known to enhance viral infectivity. Notably, only polysaccharides containing N-acetylglucosamine (GlcNAc) have demonstrated the ability to facilitate infection, rendering poliovirus more stable and increasing its overall infectivity (15). It has been observed that sulfated polysaccharides can interfere with the binding of the Severe acute respiratory syndrome coronavirus 2 (SARS-CoV-2) spike protein to the angiotensin-converting enzyme 2 (ACE2) receptor, thus preventing viral infection (16). Moreover, certain bacterial components such as fucose, galactose, and mannose have been reported to possess virucidal activity against Enterovirus 71 (17).

Intestinal microbes have a direct impact on viral infections, while they also indirectly influence them by affecting interferon (IFN) production and the intestinal barrier. For instance, when antibiotics are used, they trigger the production of IFN-λ, which has antiviral properties and prevents norovirus persistence (18). This suggests that intestinal microbes inhibit IFNs and facilitate norovirus infection. Additionally, bacterial lipopolysaccharides bind to MMTV or trigger Toll-like receptor 4 (TLR4), which induces the production of inhibitory cytokine IL-10, leading to immunological tolerance and increased MMTV invasion efficiency (19). On the other hand, certain metabolites produced by intestinal microbes can protect the host from viral infections by increasing the IFNs production and enhancing the repair ability of the intestinal barrier. In mice, oral administration of acetate has been shown to elevate IFNs in the lungs, resulting in reduced viral loads of respiratory syncytial virus (RSV) and pulmonary inflammation. These antiviral effects depend on the activation of the metabolite sensor G-protein-coupled receptor 43 (GPR43), which induces an IFN-β response (20). Another metabolite, retinoic acid, has been found to increase the abundance of *Lactobacillaceae* families, which in turn increases IFN-β levels in the macrophage cell line RAW264.7, thereby interfering with norovirus infection (21). In addition to IFNs, improved repair ability of the intestinal barrier also protects against rotavirus infection. Studies on mice have shown that *Segmented filamentous bacteria* (SFB) protect against rotavirus infection by accelerating the turnover of epithelial cells (22). In conclusion, intestinal microbes can directly influence viral infections by modulating viral infectivity and stability, or indirectly by affecting IFNs and the intestinal barrier.

## 3 Microbes may affect PEDV infection via changed intestinal barrier in piglets

Although we found that oral administration of *lactic acid bacteria* inhibits PEDV infection, there is a lack of conclusive evidence demonstrating that intestinal bacteria or their components have an impact on PEDV infection through direct contact (23). Based on the aforementioned analysis, it can be inferred that the indirect influences of intestinal microbes on PEDV infection are also mediated by IFNs and the intestinal barrier. Since piglets with the highest mortality rate exhibit compromised immune systems and reduced levels of IFNs (24), we have initiated an investigation into the potential of intestinal microbes to impede PEDV infection through their impact on the intestinal barrier (**Figure 1C**).

Severe permeability in the intestinal barrier has been reported to promote infectious diseases (25). The significant reduction of goblet cells, which are responsible for the secretion of mucin and the maintenance of intestinal integrity, in the jejunum and ileum during PEDV infection results in a damaged mucus layer and an increased vulnerability to secondary infections (26). An *in vitro* study has revealed that the methylation of the mC-5 site inhibits the expression of mucin 2, which in turn increases the susceptibility of piglets to PEDV by reducing the protective barrier (27). Additionally, pyroptosis is a type of pro-inflammatory cell death that is triggered by the gasdermin family proteins. This process involves the formation of pores on cells, the recognition of danger signals, and the release of pro-inflammatory cytokines such as IL-1β and IL-18 (28). Gasdermin D (GSDMD), a key executor of pyroptosis, has been found to play a role in safeguarding host cells from PEDV infection (29). Last but not least, GLP-2, a specific hormone that promotes intestinal growth, has been found to enhance the expression of tight junction proteins, facilitate mucosal repair, and improve the function of the intestinal barrier (30–32). Silencing the GLP-2 gene with shRNA transfection prior to infection has been shown to significantly increase the copies of PEDV in cells, indicating that a damaged barrier can facilitate PEDV infection (33).

Currently, numerous microbes have been documented as safeguarding the integrity of the intestinal barrier. *Akkermansia muciniphila* produces a pilus-like protein called Amuc_1100, which plays a crucial role in maintaining the immune homeostasis of the intestinal mucosa and improving the function of the intestinal barrier (34). In studies conducted on mice and Caco-2 cells, it was observed that the probiotic bacterium *Lactobacilli rhamnosus GG* promotes cell renewal and enhances mucosal repair following DSS-induced colitis by generating reactive oxygen species in epithelial cells (35, 36). Additionally, the extracellular proteins secreted by *Lactobacillus plantarum BMCM12* weaken the adhesion of pathogens and protect the intestinal barrier (37). Commensal microbes, including *Faecalibacterium prausnitzii*, *Roseburia intestinalis*, *Bacteroides faecis* and *Lactobacillus* have the potential to limit injury caused by inflammatory responses and improve the integrity of the epithelial barrier. Studies using *in vitro* models, specifically Caco-2 and HT29-MTX cells, have demonstrated that these four bacterial strains are capable of restoring the impaired barrier function caused by inflammatory cytokines IL-1β, TNF-α, IFN-γ, and LPS (38, 39). In addition to the intestinal microbes themselves, intestinal microbial metabolites regulate the integrity of the intestinal barrier (40). These metabolites, including indole derivatives (41), bile acid metabolites (42), conjugated fatty acids (43), polyamines (44, 45), polyphenolic derivatives (46) and short-chain fatty acids (SCFAs) (47), increase longevity, promote the recovery of injured mucosa, and have favorable effects on the intestinal barrier.

Therefore, it has been determined that the intestinal microbiota has the ability to impact the intestinal barrier, which in turn plays a crucial role in the progression of PEDV infection.

## 4 Dysbiosis of the intestinal microbes in PEDV infection

To gain novel insights into the impact of specific microbes on PEDV infection, we undertook a thorough review of the published literature (48–52) and narrowed our focus to those microbes that exhibited significant changes following PEDV infection (**Figure 1D**). Following classification by generic name, bacterial features, pathogenic characteristics, and immune function, we compiled a representative list of bacteria that could potentially influence PEDV infection, which is presented in **Table 1**.

**Table 1:** Bacterial change after PEDV infection and corresponding bacterial feature.

Bacterium	Morphological staining	Pathogenic substance	Biochemical characteristics, immunologic function
*Bacteroides* ↓	G-, mostly no flagella, no spore, coccobacilli.	Capsular polysaccharide, lipopolysaccharide, toxin, enzyme	Facultatively anaerobic, SCFAs producing bacteria; T cell-dependent immune response protect against abscess (53).
*Clostridium butyricum* ↓	G+, flagella, spore, Cocci.	Botulinum toxin type E	Ability to interfere with the growth of commensal bacteria, produce SCFAs, mainly butyrate, contributing to intestinal health, improve mucosal immunity (54, 55).
*Psychrobacter* ↓	G-, no flagella, no spore, coccobacilli.	Hypo-acylated lipopolysaccharide	Aerobic, improve the numbers of probiotics, enhance digestive efficiency and innate immunity (56, 57).
*Enterococcus* ↑	G+, flagella, no spore, coccobacilli	Surface protein, cytolysin, collagen-binding protein, aggregation substance, endocarditis antigen, gelatinase, capsular polysaccharide, hyaluronidase (58)	Facultatively anaerobic, improve adhesion (59), modulate inflammatory response (60).
*Fusobacterium* ↑	G-, no flagella, no spore, pleomorphism	Hemagglutinin, hemolysin, lipopolysaccharide, leukotoxin, collagenolytic cell wall component.	Anaerobic, cause tissue necrosis and septicemia (61), cause intestinal inflammation (62).
*Escherichia* ↑	G-, flagella, no spores, capsule, fimbriae, rod	Adhesin, colonization factor antigen, fimbriae, exotoxin, enterotoxin, Shiga toxin, lipid A of lipoid polysaccharide	Facultatively anaerobic, decrease phagocytosis, prevent cell cycle, destroy cellular junctions, inhibit IFNs, enhance adhesion, induce apoptosis and inflammation (63).
*Desulfovibrionaceae* ↑	G-, no spores, monopole hair, coccobacilli.	Tetrodotoxin	Facultatively anaerobic, induce intestinal dysbacteriosis, inflammation, and disrupt intestinal barrier function (64).

The presented **Table 1** indicates a decrease in the presence of bacteria associated with intestinal health, such as *Bacteroides*, *Clostridium butyricum*, and *Psychrobacter*, during PEDV infection. Conversely, there was an increase in the presence of bacteria like *Enterococcus*, *Fusobacterium*, *Escherichia*, and *Desulfovibrionaceae*. Notably, *Bacteroides* (including *Prevotella*), *Clostridium butyricum*, and *Clostridium leptum* (including *Faecalibacterium*) are known to secrete SCFAs (53–55). These SCFAs have been shown to protect intestinal integrity through GPRs, aid in the repair of damaged intestinal mucosa, and mitigate inflammation-induced damage (65, 66). *Clostridium butyricum* has the capability to augment the population of commensal bacteria including *Bifidobacteria*, suppress the proliferation of pathogenic bacteria like *Shigella dysenteriae*, reestablish the equilibrium of gut microbes, diminish the generation of enterotoxins that are toxic to intestinal mucosa such as amines, ammonia, and indoles, reinstate intestinal immune function, and regulate normal physiological function (54, 55). *Psychrobacter* enhances the number of probiotics, elevates digestive efficiency, and fortifies innate immunity through the TLR-mediated pathway (56, 57).

Meanwhile, certain bacteria become increased in the intestines following PEDV infection, potentially causing further harm. For instance, *Enterococcus* has been known to cause inflammation and a range of infections in humans, such as urinary tract infections, bacterial endocarditis, and meningitis (67). *Fusobacteria* secrete leukotoxin, which can impede the body’s ability to clear bacteria and lead to tissue destruction (68). This type of bacteria has also been linked to intestinal tumors, acute appendicitis, and sepsis (62). *Proteobacteria*, which include *Escherichia* and *Desulfovibrionaceae*, have been shown to suppress mucosal immunity, disrupt the cell cycle, and cause DNA damage in the intestines. Specifically, *E. coli* strains such as *Enteropathogenic Escherichia coli (EPEC)* have been found to interfere with phagocytosis, disrupt cellular trafficking, induce apoptosis, and damage cellular junctions. *Enterotoxigenic Escherichia coli (ETEC)*, on the other hand, inhibits the production of antimicrobial peptides and binds closely to host cells through flagella and outer-membrane proteins (63). Finally, *Desulfovibrionaceae* and the genus *Desulfovibrio* have been shown to disrupt butyrate oxidation, cause intestinal dysbiosis and inflammation, and damage the intestinal barrier function (64).

## 5 Speculative bacterial factors influencing PEDV invasion

In accordance with the earlier paragraph, the microbes that exhibit changes following PEDV infection harbor the potential to affect viral infection by influencing the intestinal barrier. Given the common characteristics of these microbes, it was conjectured that SCFAs, bacterial cell components, and toxins originating from pathogenic bacteria could contribute to the pathogenesis of PEDV infection (**Figure 1E**).

The primary source of SCFAs is the anaerobic fermentation of undigested carbohydrates in the intestines, which results in the production of acetic acid, propionic acid, and butyric acid. Metagenomic analysis has revealed that acetate production pathways are widely distributed among bacteria and are most concentrated in the intestinal gut (69). Conversely, propionate and butyrate production pathways are more conserved and substrate-specific (70). SCFAs play a crucial role in cell metabolism and the growth of mucosal cells in the intestine. They also have anti-inflammatory properties and help to reduce damage to the intestine (71). The colonization of germ-free mice with SCFAs-producing *Bacteroides thetaiotaomicron* or *Faecalibacterium prausnitzii* resulted in the differentiation of goblet cells and production of mucus *in vivo* (72). Similarly, in an *in vitro* system simulating the mucus- and lumen-associated microbes, supplementation with butyrate-producing bacteria, *Butyrococcus pullicaecorum*, improved epithelial integrity and sustained intestinal barrier via butyrate in Crohn’s disease patients (73). High concentrations of SCFAs increased tight junction function and *Bifidobacterium* abundance, thereby improving the intestinal barrier and protecting against *enteropathogenic Escherichia coli* O157:H7 infection (74). SCFAs also exhibited anti-inflammatory effects by inhibiting the expression of TNF-α and IL-6 in IFN-γ-stimulated RAW 264.7 cells (75). Thus, increasing the production of SCFAs may alleviate PEDV infection and its associated damage by inhibiting inflammation and strengthening the intestinal barrier.

Furthermore, certain components present in bacteria cells, such as glycan and flagellin, may facilitate viral infection. Studies have shown that the removal of coated sugars on the surface of the virus through neuraminidase treatment resulted in a reduction in the binding efficiency of PEDV (76). This highlights the significance of glycan structure in the invasion of PEDV, as the spike protein of coronaviruses has also been found to bind to glycan-containing mucins to aid in viral invasion (77, 78). Additionally, flagella and the complex network of glycans on the surface of bacteria, including the peptidoglycan layer, lipoteichoic acids, and lipopolysaccharides (79), have the potential to increase viral infection by binding to viruses and enhancing virion stability, although their precise role in this process remains unclear (80). In addition, the health of the intestines is also impacted by bacterial toxins (81). LPS, as the primary toxic component of endotoxin, augments the adhesion and stability of the virus, thereby facilitating the invasion of poliovirus (82). Apart from its ability to adhere, LPS can cause inflammation-related damage to the intestinal barrier, which increases the likelihood of PEDV infection (83). Besides, the toxin produced by *Shigella* has potent enterotoxicity, leading to cytoskeleton rearrangement, increased permeability, and facilitating viral invasion (84).

Upon examining the frequency of microbes before and after PEDV infection, it can be inferred that SCFAs have the ability to safeguard the intestinal barrier and impede PEDV invasion, whereas some bacterial components and secreted toxins have an adverse impact.

## 6 Perspectives

Thus, our proposal is to amplify the microbial population in the intestines that produce SCFAs. This will heighten the intestinal barrier’s integrity and present a new approach to developing drugs targeting PEDV infection. The production of propionate and butyrate in the human intestine involves various pathways utilized by different bacterial species. *Bacteroidetes* and *Negativicutes* use the succinate pathway, while *Lachnospiraceae* use the propanediol pathway to produce propionate (69). *Akkermansia municiphilla* has also been identified as a producer of propionate through the degradation of mucus in the human intestine (85). Butyrate is mainly produced by *Ruminococcus bromii* (86), *Faecalibacterium prausnitzii*, *Eubacterium rectale*, and *Eubacterium hallii*, which ferment resistant starch in the colon (87). The majority of butyrate producers utilize the butyryl CoA:acetate CoA transferase pathway, while only a few, such as *Coprococcus eutactus*, use the butyrate kinase route (70, 88, 89). Besides, *Butyricicoccus*, *Roseburia*, *Lachnospiraceae*, *Rikenella*, and *Eubacterium xylanophilum* are also listed as SCFAs-producing bacteria (90).

In order to optimize the fermentation process for the production of SCFAs through microbial activity, it is essential to carefully monitor and control the pH, iron levels, and oxygen concentration. For instance, when comparing the abilities of *Firmicutes* and *Bacteroides*, it can be observed that the latter has a lower capacity to adapt to a pH of 5.5, which can significantly limit the production of propionate and butyrate (91). The scarcity of iron in mice causes a decline in the number of SCFAs-producing bacteria, including *Eubacterium rectale*, which leads to a reduction in the concentration of propionate and butyrate. Nevertheless, the addition of FeSO_4_ to the mice reinstates the microbial abundance and butyrate concentration (92). The presence of oxygen is not a crucial factor for the growth of *Faecalibacterium prausnitzii*, an obligate anaerobe, as it flourishes optimally in low oxygen concentration, but not in an oxygen-deprived environment (93).

Currently, the prevailing technique utilized to alter the constitution of intestinal microbiota is Fecal Microbial Transplantation (FMT) (94). FMT has been shown to be effective in treating inflammatory bowel diseases, metabolic diseases, autoimmune diseases, and even cardiovascular diseases. According to a large retrospective study, FMT has been proven to be both effective and safe in treating *Clostridium difficile* infection in children and young adults (95). However, to investigate the hypothesis that the colonization of probiotics in the intestine can prevent PEDV infection, it is imperative to conduct both *in vitro* virus infected cell experiments and *in vivo* pig experiments (**Figure 1F**). Additionally, it is necessary to devise strategies to efficiently colonize probiotics in the intestine and enhance the distribution of microbes to prevent PEDV infection.

## 7 Conclusion

As shown in **Figure 1**, this review highlights the potential of intestinal microbes to influence PEDV infection by impacting the intestinal barrier in piglets. By analyzing the dysbiosis of microbes following PEDV infection, it is evident that increasing the production of SCFA by bacteria could potentially inhibit viral infection. However, further evidence is required to substantiate the claim that modulating microbes can inhibit PEDV infection.

## Author contributions

SY and GL conceived the project. SY analyzed the data and drafted the manuscript. CJ sorted out the structure and provide some new ideas. SY, GL. HS, CJ and BL revised the language and edited the manuscript. All authors contributed to the article and approved the submitted version.

## References

[1] Kocherhans R Bridgen A Ackermann M Tobler K. Completion of the porcine epidemic diarrhoea coronavirus (PEDV) genome sequence. Virus Genes (2001) 23(2):137–44. doi: 10.1023/A:1011831902219 PMC7089135 11724265

[2] Lin CN Chung WB Chang SW Wen CC Liu H Chien CH. US-Like strain of porcine epidemic diarrhea virus outbreaks in Taiwan, 2013-2014. J Vet Med Sci (2014) 76(9):1297–9. doi: 10.1292/jvms.14-0098 PMC4197162 24898162

[3] Wang XM Niu BB Yan H Gao DS Yang X Chen L. Genetic properties of endemic Chinese porcine epidemic diarrhea virus strains isolated since 2010. Arch Virol (2013) 158(12):2487–94. doi: 10.1007/s00705-013-1767-7 PMC7087078 23797760

[4] Sergeev OV. [Porcine epidemic diarrhea]. Vopr Virusol. (2009) 54(2):4–8. 19459405

[5] Ali A Tan H Kaiko GE. Role of the intestinal epithelium and its interaction with the microbiota in food allergy. Front Immunol (2020) 11:604054. doi: 10.3389/fimmu.2020.604054 33365031 PMC7750388

[6] Vancamelbeke M Vermeire S. The intestinal barrier: a fundamental role in health and disease. Expert Rev Gastroenterol Hepatol (2017) 11(9):821–34. doi: 10.1080/17474124.2017.1343143 PMC6104804 28650209

[7] Kamada N Seo SU Chen GY Nunez G. Role of the gut microbiota in immunity and inflammatory disease. Nat Rev Immunol (2013) 13(5):321–35. doi: 10.1038/nri3430 23618829

[8] Yang M Yang Y He Q Zhu P Liu M Xu J. Intestinal microbiota-a promising target for antiviral therapy? Front Immunol (2021) 12:676232. doi: 10.3389/fimmu.2021.676232 34054866 PMC8149780

[9] Erickson AK Jesudhasan PR Mayer MJ Narbad A Winter SE Pfeiffer JK. Bacteria facilitate enteric virus Co-infection of mammalian cells and promote genetic recombination. Cell Host Microbe (2018) 23(1):77–88 e5. doi: 10.1016/j.chom.2017.11.007 29290575 PMC5764776

[10] Jones MK Watanabe M Zhu S Graves CL Keyes LR Grau KR. Enteric bacteria promote human and mouse norovirus infection of B cells. Science (2014) 346(6210):755–9. doi: 10.1126/science.1257147 PMC4401463 25378626

[11] Esseili MA Gao X Boley P Hou Y Saif LJ Brewer-Jensen P. Human norovirus histo-blood group antigen (HBGA) binding sites mediate the virus specific interactions with lettuce carbohydrates. Viruses (2019) 11(9):883. doi: 10.3390/v11090833 PMC6784273 31500340

[12] Li D Breiman A le Pendu J Uyttendaele M. Binding to histo-blood group antigen-expressing bacteria protects human norovirus from acute heat stress. Front Microbiol (2015) 6:659. doi: 10.3389/fmicb.2015.00659 26191052 PMC4486850

[13] Miura T Sano D Suenaga A Yoshimura T Fuzawa M Nakagomi T. Histo-blood group antigen-like substances of human enteric bacteria as specific adsorbents for human noroviruses. J Virol (2013) 87(17):9441–51. doi: 10.1128/JVI.01060-13 PMC3754087 23804639

[14] Almand EA Moore MD Outlaw J Jaykus LA. Human norovirus binding to select bacteria representative of the human gut microbiota. PloS One (2017) 12(3):e0173124. doi: 10.1371/journal.pone.0173124 28257478 PMC5336261

[15] Kuss SK Best GT Etheredge CA Pruijssers AJ Frierson JM Hooper LV. Intestinal microbiota promote enteric virus replication and systemic pathogenesis. Science (2011) 334(6053):249–52. doi: 10.1126/science.1211057 PMC3222156 21998395

[16] Barbosa JR de Fatima Henriques Lourenco L. Sulfated polysaccharides act as baits to interfere with the binding of the spike protein (SARS-CoV-2) to the ACE2 receptor and can be administered through food. J Funct Foods (2023) 104:105532. doi: 10.1016/j.jff.2023.105532 37035109 PMC10073580

[17] Li Z Cui B Liu X Wang L Xian Q Lu Z. Virucidal activity and the antiviral mechanism of acidic polysaccharides against enterovirus 71 infection *in vitro* . Microbiol Immunol (2020) 64(3):189–201. doi: 10.1111/1348-0421.12763 31785100

[18] Baldridge MT Nice TJ McCune BT Yokoyama CC Kambal A Wheadon M. Commensal microbes and interferon-lambda determine persistence of enteric murine norovirus infection. Science (2015) 347(6219):266–9. doi: 10.1126/science.1258025 PMC4409937 25431490

[19] Kane M Case LK Kopaskie K Kozlova A MacDearmid C Chervonsky AV. Successful transmission of a retrovirus depends on the commensal microbiota. Science (2011) 334(6053):245–9. doi: 10.1126/science.1210718 PMC3519937 21998394

[20] Antunes KH Fachi JL de Paula R da Silva EF Pral LP Dos Santos AA. Microbiota-derived acetate protects against respiratory syncytial virus infection through a GPR43-type 1 interferon response. Nat Commun (2019) 10(1):3273. doi: 10.1038/s41467-019-11152-6 31332169 PMC6646332

[21] Lee H Ko G. Antiviral effect of vitamin a on norovirus infection via modulation of the gut microbiome. Sci Rep (2016) 6:25835. doi: 10.1038/srep25835 27180604 PMC4867650

[22] Shi Z Zou J Zhang Z Zhao X Noriega J Zhang B. Segmented filamentous bacteria prevent and cure rotavirus infection. Cell (2019) 179(3):644–58 e13. doi: 10.1016/j.cell.2019.09.028 31607511 PMC7525827

[23] Yang S Li S Lu Y Jansen CA Savelkoul HFJ Liu G. Oral administration of lactic acid bacteria inhibits PEDV infection in young piglets. Virology (2023) 579:1–8. doi: 10.1016/j.virol.2022.12.005 36584644

[24] Yang S Yang N Huang X Li Y Liu G Jansen CA. Pigs’ intestinal barrier function is more refined with aging. Dev Comp Immunol (2022) 136:104512. doi: 10.1016/j.dci.2022.104512 35995250

[25] Iacob S Iacob DG. Infectious threats, the intestinal barrier, and its Trojan horse: dysbiosis. Front Microbiol (2019) 10:1676. doi: 10.3389/fmicb.2019.01676 31447793 PMC6692454

[26] Jung K Saif LJ. Goblet cell depletion in small intestinal villous and crypt epithelium of conventional nursing and weaned pigs infected with porcine epidemic diarrhea virus. Res Vet Sci (2017) 110:12–5. doi: 10.1016/j.rvsc.2016.10.009 28159230

[27] Xiao Y Zhou Y Sun S Wang H Wu S Bao W. Effect of promoter methylation on the expression of porcine MUC2 gene and resistance to PEDV infection. Front Vet Sci (2021) 8:646408. doi: 10.3389/fvets.2021.646408 33996974 PMC8116951

[28] Zhang S Liang Y Yao J Li DF Wang LS. Role of pyroptosis in inflammatory bowel disease (IBD): from gasdermins to DAMPs. Front Pharmacol (2022) 13:833588. doi: 10.3389/fphar.2022.833588 35677444 PMC9168461

[29] Shi F Lv Q Wang T Xu J Xu W Shi Y. Coronaviruses Nsp5 antagonizes porcine gasdermin d-mediated pyroptosis by cleaving pore-forming p30 fragment. mBio (2022) 13(1):e0273921. doi: 10.1128/mbio.02739-21 35012343 PMC8749417

[30] Moran GW O’Neill C McLaughlin JT. GLP-2 enhances barrier formation and attenuates TNFα-induced changes in a caco-2 cell model of the intestinal barrier. Regul Peptides (2012) 178(1-3):95–101. doi: 10.1016/j.regpep.2012.07.002 22809889

[31] Benjamin MA McKay DM Yang PC Cameron H Perdue MH. Glucagon-like peptide-2 enhances intestinal epithelial barrier function of both transcellular and paracellular pathways in the mouse. Gut (2000) 47(1):112–9. doi: 10.1136/gut.47.1.112 PMC1727982 10861272

[32] Petersen YM Hartmann B Holst JJ Le Huerou-Luron I Bjornvad CR Sangild PT. Introduction of enteral food increases plasma GLP-2 and decreases GLP-2 receptor mRNA abundance during pig development. J Nutr (2003) 133(6):1781–6. doi: 10.1093/jn/133.6.1781 12771317

[33] Zhou Y Ren Z Zhang S Wang H Wu S Bao W. Analysis of intestinal mucosa integrity and GLP-2 gene functions upon porcine epidemic diarrhea virus infection in pigs. Anim (Basel) (2021) 11(3):644. doi: 10.3390/ani11030644 PMC8000733 33804466

[34] Ottman N Reunanen J Meijerink M Pietila TE Kainulainen V Klievink J. Pili-like proteins of akkermansia muciniphila modulate host immune responses and gut barrier function. PloS One (2017) 12(3):e0173004. doi: 10.1371/journal.pone.0173004 28249045 PMC5332112

[35] Swanson PA 2nd Kumar A Samarin S Vijay-Kumar M Kundu K Murthy N. Enteric commensal bacteria potentiate epithelial restitution via reactive oxygen species-mediated inactivation of focal adhesion kinase phosphatases. Proc Natl Acad Sci United States America (2011) 108(21):8803–8. doi: 10.1073/pnas.1010042108 PMC3102402 21555563

[36] Ohland CL Macnaughton WK. Probiotic bacteria and intestinal epithelial barrier function. Am J Physiol Gastrointest Liver Physiol (2010) 298(6):G807–19. doi: 10.1152/ajpgi.00243.2009 20299599

[37] Liu Q Yu Z Tian F Zhao J Zhang H Zhai Q. Surface components and metabolites of probiotics for regulation of intestinal epithelial barrier. Microb Cell Fact (2020) 19(1):23. doi: 10.1186/s12934-020-1289-4 32024520 PMC7003451

[38] Mohebali N Ekat K Kreikemeyer B Breitruck A. Barrier protection and recovery effects of gut commensal bacteria on differentiated intestinal epithelial cells *in vitro* . Nutrients (2020) 12(8):2251. doi: 10.3390/nu12082251 PMC7468801 32731411

[39] Chen Y Cui W Li X Yang H. Interaction between commensal bacteria, immune response and the intestinal barrier in inflammatory bowel disease. Front Immunol (2021) 12:761981. doi: 10.3389/fimmu.2021.761981 34858414 PMC8632219

[40] Li Z Quan G Jiang X Yang Y Ding X Zhang D. Effects of metabolites derived from gut microbiota and hosts on pathogens. Front Cell Infect Microbiol (2018) 8:314. doi: 10.3389/fcimb.2018.00314 30276161 PMC6152485

[41] Shimada Y Kinoshita M Harada K Mizutani M Masahata K Kayama H. Commensal bacteria-dependent indole production enhances epithelial barrier function in the colon. PloS One (2013) 8(11):e80604. doi: 10.1371/journal.pone.0080604 24278294 PMC3835565

[42] Cipriani S Mencarelli A Chini MG Distrutti E Renga B Bifulco G. The bile acid receptor GPBAR-1 (TGR5) modulates integrity of intestinal barrier and immune response to experimental colitis. PloS One (2011) 6(10):e25637. doi: 10.1371/journal.pone.0025637 22046243 PMC3203117

[43] Chen Y Yang B Ross RP Jin Y Stanton C Zhao J. Orally administered CLA ameliorates DSS-induced colitis in mice via intestinal barrier improvement, oxidative stress reduction, and inflammatory cytokine and gut microbiota modulation. J Agric Food Chem (2019) 67(48):13282–98. doi: 10.1021/acs.jafc.9b05744 31690068

[44] Tofalo R Cocchi S Suzzi G. Polyamines and gut microbiota. Front Nutr (2019) 6:16. doi: 10.3389/fnut.2019.00016 30859104 PMC6397830

[45] Timmons J Chang ET Wang JY Rao JN. Polyamines and gut mucosal homeostasis. J Gastrointest Dig Syst (2012) 2(Suppl 7). doi: 10.4172/2161-069X.S7-001 PMC4165078 25237589

[46] Johnson SL Kirk RD DaSilva NA Ma H Seeram NP Bertin MJ. Polyphenol microbial metabolites exhibit gut and blood(-)Brain barrier permeability and protect murine microglia against LPS-induced inflammation. Metabolites (2019) 9(4):78. doi: 10.3390/metabo9040078 PMC6523162 31010159

[47] Akhtar M Chen Y Ma Z Zhang X Shi D Khan JA. Gut microbiota-derived short chain fatty acids are potential mediators in gut inflammation. Anim Nutr (2022) 8:350–60. doi: 10.1016/j.aninu.2021.11.005 PMC9040132 35510031

[48] Huang MZ Wang SY Wang H Cui DA Yang YJ Liu XW. Differences in the intestinal microbiota between uninfected piglets and piglets infected with porcine epidemic diarrhea virus. PloS One (2018) 13(2):e0192992. doi: 10.1371/journal.pone.0192992 29447243 PMC5814011

[49] Koh HW Kim MS Lee JS Kim H Park SJ. Changes in the swine gut microbiota in response to porcine epidemic diarrhea infection. Microbes Environ (2015) 30(3):284–7. doi: 10.1264/jsme2.ME15046 PMC4567570 26212519

[50] Liu S Zhao L Zhai Z Zhao W Ding J Dai R. Porcine epidemic diarrhea virus infection induced the unbalance of gut microbiota in piglets. Curr Microbiol (2015) 71(6):643–9. doi: 10.1007/s00284-015-0895-6 26319658

[51] Song D Peng Q Chen Y Zhou X Zhang F Li A. Altered gut microbiota profiles in sows and neonatal piglets associated with porcine epidemic diarrhea virus infection. Sci Rep (2017) 7(1):17439. doi: 10.1038/s41598-017-17830-z 29234140 PMC5727058

[52] Tan Z Dong W Ding Y Ding X Zhang Q Jiang L. Changes in cecal microbiota community of suckling piglets infected with porcine epidemic diarrhea virus. PloS One (2019) 14(7):e0219868. doi: 10.1371/journal.pone.0219868 31310635 PMC6634403

[53] Onderdonk AB Markham RB Zaleznik DF Cisneros RL Kasper DL. Evidence for T cell-dependent immunity to bacteroides fragilis in an intraabdominal abscess model. J Clin Invest (1982) 69(1):9–16. doi: 10.1172/JCI110445 6976357 PMC371162

[54] Chen L Li S Zheng J Li W Jiang X Zhao X. Effects of dietary clostridium butyricum supplementation on growth performance, intestinal development, and immune response of weaned piglets challenged with lipopolysaccharide. J Anim Sci Biotechnol (2018) 9:62. doi: 10.1186/s40104-018-0275-8 30159141 PMC6106813

[55] Hamer HM Jonkers D Venema K Vanhoutvin S Troost FJ Brummer RJ. Review article: the role of butyrate on colonic function. Aliment Pharmacol Ther (2008) 27(2):104–19. doi: 10.1111/j.1365-2036.2007.03562.x 17973645

[56] Korneev KV Kondakova AN Arbatsky NP Novototskaya-Vlasova KA Rivkina EM Anisimov AP. Distinct biological activity of lipopolysaccharides with different lipid a acylation status from mutant strains of yersinia pestis and some members of genus psychrobacter. Biochem (Mosc) (2014) 79(12):1333–8. doi: 10.1134/S0006297914120062 25716726

[57] Sun Y Yang HL Ma RL Zhang C Lin WY. Effect of dietary administration of psychrobacter sp. on the growth, feed utilization, digestive enzymes and immune responses of grouper epinephelus coioides. Aquaculture Nutr (2010) 17:e733–e40. doi: 10.1111/j.1365-2095.2010.00837.x

[58] Zou LK Wang HN Zeng B Li JN Li XT Zhang AY. Erythromycin resistance and virulence genes in enterococcus faecalis from swine in China. New Microbiol (2011) 34(1):73–80. 21344149

[59] Leitch EC Walker AW Duncan SH Holtrop G Flint HJ. Selective colonization of insoluble substrates by human faecal bacteria. Environ Microbiol (2007) 9(3):667–79. doi: 10.1111/j.1462-2920.2006.01186.x 17298367

[60] Wang S Ng LH Chow WL Lee YK. Infant intestinal enterococcus faecalis down-regulates inflammatory responses in human intestinal cell lines. World J Gastroenterol (2008) 14(7):1067–76. doi: 10.3748/wjg.14.1067 PMC2689410 18286689

[61] Narayanan SK Nagaraja TG Chengappa MM Stewart GC. Cloning, sequencing, and expression of the leukotoxin gene from fusobacterium necrophorum. Infect Immun (2001) 69(9):5447–55. doi: 10.1128/IAI.69.9.5447-5455.2001 PMC98656 11500416

[62] Jia YP Wang K Zhang ZJ Tong YN Han D Hu CY. TLR2/TLR4 activation induces tregs and suppresses intestinal inflammation caused by fusobacterium nucleatum *in vivo* . PloS One (2017) 12(10):e0186179. doi: 10.1371/journal.pone.0186179 29016688 PMC5633168

[63] Croxen MA Finlay BB. Molecular mechanisms of escherichia coli pathogenicity. Nat Rev Microbiol (2010) 8(1):26–38. doi: 10.1038/nrmicro2265 19966814

[64] Gill SR Pop M Deboy RT Eckburg PB Turnbaugh PJ Samuel BS. Metagenomic analysis of the human distal gut microbiome. Science (2006) 312(5778):1355–9. doi: 10.1126/science.1124234 PMC3027896 16741115

[65] Jiang H Ling Z Zhang Y Mao H Ma Z Yin Y. Altered fecal microbiota composition in patients with major depressive disorder. Brain Behav Immun (2015) 48:186–94. doi: 10.1016/j.bbi.2015.03.016 25882912

[66] Miquel S Martin R Rossi O Bermudez-Humaran LG Chatel JM Sokol H. Faecalibacterium prausnitzii and human intestinal health. Curr Opin Microbiol (2013) 16(3):255–61. doi: 10.1016/j.mib.2013.06.003 23831042

[67] Fisher K Phillips C. The ecology, epidemiology and virulence of enterococcus. Microbiol (Reading) (2009) 155(Pt 6):1749–57. doi: 10.1099/mic.0.026385-0 19383684

[68] Ramos HC Rumbo M Sirard JC. Bacterial flagellins: mediators of pathogenicity and host immune responses in mucosa. Trends Microbiol (2004) 12(11):509–17. doi: 10.1016/j.tim.2004.09.002 15488392

[69] Louis P Flint HJ. Formation of propionate and butyrate by the human colonic microbiota. Environ Microbiol (2017) 19(1):29–41. doi: 10.1111/1462-2920.13589 27928878

[70] Morrison DJ Preston T. Formation of short chain fatty acids by the gut microbiota and their impact on human metabolism. Gut Microbes (2016) 7(3):189–200. doi: 10.1080/19490976.2015.1134082 26963409 PMC4939913

[71] Shi P Su Y Li R Liang Z Dong S Huang J. PEDV nsp16 negatively regulates innate immunity to promote viral proliferation. Virus Res (2019) 265:57–66. doi: 10.1016/j.virusres.2019.03.005 30849413 PMC7114654

[72] Wrzosek L Miquel S Noordine ML Bouet S Joncquel Chevalier-Curt M Robert V. Bacteroides thetaiotaomicron and faecalibacterium prausnitzii influence the production of mucus glycans and the development of goblet cells in the colonic epithelium of a gnotobiotic model rodent. BMC Biol (2013) 11:61. doi: 10.1186/1741-7007-11-61 23692866 PMC3673873

[73] Geirnaert A Calatayud M Grootaert C Laukens D Devriese S Smagghe G. Butyrate-producing bacteria supplemented *in vitro* to crohn’s disease patient microbiota increased butyrate production and enhanced intestinal epithelial barrier integrity. Sci Rep (2017) 7(1):11450. doi: 10.1038/s41598-017-11734-8 28904372 PMC5597586

[74] Fukuda S Toh H Hase K Oshima K Nakanishi Y Yoshimura K. Bifidobacteria can protect from enteropathogenic infection through production of acetate. Nature (2011) 469(7331):543–7. doi: 10.1038/nature09646 21270894

[75] Park JS Lee EJ Lee JC Kim WK Kim HS. Anti-inflammatory effects of short chain fatty acids in IFN-gamma-stimulated RAW 264.7 murine macrophage cells: involvement of NF-kappaB and ERK signaling pathways. Int Immunopharmacol (2007) 7(1):70–7. doi: 10.1016/j.intimp.2006.08.015 17161819

[76] Liu C Tang J Ma Y Liang X Yang Y Peng G. Receptor usage and cell entry of porcine epidemic diarrhea coronavirus. J Virol (2015) 89(11):6121–5. doi: 10.1128/JVI.00430-15 PMC4442452 25787280

[77] Hao W Ma B Li Z Wang X Gao X Li Y. Binding of the SARS-CoV-2 spike protein to glycans. Sci Bull (Beijing) (2021) 66(12):1205–14. doi: 10.1016/j.scib.2021.01.010 PMC7816574 33495714

[78] Ryzhikov AB Onkhonova GS Imatdinov IR Gavrilova EV Maksyutov RA Gordeeva EA. Recombinant SARS-CoV-2 s protein binds to glycans of the lactosamine family *in vitro* . Biochem (Mosc) (2021) 86(3):243–7. doi: 10.1134/S0006297921030019 PMC7905424 33838626

[79] Oinam L Minoshima F Tateno H. Glycan profiling of the gut microbiota by glycan-seq. ISME Commun (2022) 2(1):1. doi: 10.1038/s43705-021-00084-2 PMC9723764 37938656

[80] Berger AK Mainou BA. Interactions between enteric bacteria and eukaryotic viruses impact the outcome of infection. Viruses (2018) 10(1):19. doi: 10.3390/v10010019 PMC5795432 29301335

[81] Chu S-HW Walker WA. Bacterial toxin interaction with the developing intestine. Gastroenterology (1993) 104(3):916–25. doi: 10.1016/0016-5085(93)91032-D 8382647

[82] Robinson CM Jesudhasan PR Pfeiffer JK. Bacterial lipopolysaccharide binding enhances virion stability and promotes environmental fitness of an enteric virus. Cell Host Microbe (2014) 15(1):36–46. doi: 10.1016/j.chom.2013.12.004 24439896 PMC3920179

[83] Guo S Al-Sadi R Said HM Ma TY. Lipopolysaccharide causes an increase in intestinal tight junction permeability *in vitro* and *in vivo* by inducing enterocyte membrane expression and localization of TLR-4 and CD14. Am J Pathol (2013) 182(2):375–87. doi: 10.1016/j.ajpath.2012.10.014 PMC3562736 23201091

[84] Duan Q Zhou M Zhu X Bao W Wu S Ruan X. The flagella of F18ab escherichia coli is a virulence factor that contributes to infection in a IPEC-J2 cell model *in vitro* . Veterinary Microbiol (2012) 160(1-2):132–40. doi: 10.1016/j.vetmic.2012.05.015 22658629

[85] Derrien M Vaughan EE Plugge CM de Vos WM. Akkermansia muciniphila gen. nov., sp. nov., a human intestinal mucin-degrading bacterium. Int J Syst Evol Microbiol (2004) 54(Pt 5):1469–76. doi: 10.1099/ijs.0.02873-0 15388697

[86] Ze X Duncan SH Louis P Flint HJ. Ruminococcus bromii is a keystone species for the degradation of resistant starch in the human colon. ISME J (2012) 6(8):1535–43. doi: 10.1038/ismej.2012.4 PMC3400402 22343308

[87] Louis P Young P Holtrop G Flint HJ. Diversity of human colonic butyrate-producing bacteria revealed by analysis of the butyryl-CoA:acetate CoA-transferase gene. Environ Microbiol (2010) 12(2):304–14. doi: 10.1111/j.1462-2920.2009.02066.x 19807780

[88] den Besten G van Eunen K Groen AK Venema K Reijngoud DJ Bakker BM. The role of short-chain fatty acids in the interplay between diet, gut microbiota, and host energy metabolism. J Lipid Res (2013) 54(9):2325–40. doi: 10.1194/jlr.R036012 PMC3735932 23821742

[89] Parada Venegas D de la Fuente MK Landskron G Gonzalez MJ Quera R Dijkstra G. Corrigendum: short chain fatty acids (SCFAs)-mediated gut epithelial and immune regulation and its relevance for inflammatory bowel diseases. Front Immunol (2019) 10:1486. doi: 10.3389/fimmu.2019.01486 31316522 PMC6611342

[90] Zhou Y Xu H Xu J Guo X Zhao H Chen Y. F. prausnitzii and its supernatant increase SCFAs-producing bacteria to restore gut dysbiosis in TNBS-induced colitis. AMB Express (2021) 11(1):33. doi: 10.1186/s13568-021-01197-6 33641084 PMC7914335

[91] Walker AW Duncan SH McWilliam Leitch EC Child MW Flint HJ. pH and peptide supply can radically alter bacterial populations and short-chain fatty acid ratios within microbial communities from the human colon. Appl Environ Microbiol (2005) 71(7):3692–700. doi: 10.1128/AEM.71.7.3692-3700.2005 PMC1169066 16000778

[92] Dostal A Chassard C Hilty FM Zimmermann MB Jaeggi T Rossi S. Iron depletion and repletion with ferrous sulfate or electrolytic iron modifies the composition and metabolic activity of the gut microbiota in rats. J Nutr (2012) 142(2):271–7. doi: 10.3945/jn.111.148643 PMC3260059 22190022

[93] Khan MT Duncan SH Stams AJ van Dijl JM Flint HJ Harmsen HJ. The gut anaerobe faecalibacterium prausnitzii uses an extracellular electron shuttle to grow at oxic-anoxic interphases. ISME J (2012) 6(8):1578–85. doi: 10.1038/ismej.2012.5 PMC3400418 22357539

[94] Borody T. Chapter 18 - fecal microbiota transplantation: treatment of the gut microbiome. In: Eslick GD, editor. Gastrointestinal diseases and their associated infections. Elsevier: Philadelphia (2019). p. 249–61.

[95] Nicholson MR Mitchell PD Alexander E Ballal S Bartlett M Becker P. Efficacy of fecal microbiota transplantation for clostridium difficile infection in children. Clin Gastroenterol Hepatol (2020) 18(3):612–9 e1. doi: 10.1016/j.cgh.2019.04.037 31009795 PMC7549313

